# Integrating surgical intervention and watch-and-wait approach in dMMR metastatic rectal cancer with pembrolizumab: a case report

**DOI:** 10.1186/s40792-024-01994-8

**Published:** 2024-08-26

**Authors:** Yohei Ando, Tsubasa Sakurai, Kosuke Ozaki, Shimpei Matsui, Toshiki Mukai, Tomohiro Yamaguchi, Takashi Akiyoshi, Izuma Nakayama, Yasuyuki Shigematsu, Atsushi Oba, Akiko Chino, Yosuke Fukunaga

**Affiliations:** 1 Department of Gastroenterological Surgery, Cancer Institute Hospital of the Japanese Foundation for Cancer Research, 3-8-31 Ariake, Koto-Ku, Tokyo, 135-8550 Japan; 2 Department of Gastroenterological Chemotherapy, Cancer Institute Hospital of Japanese Foundation for Cancer Research, 3-8-31 Ariake, Koto-Ku, Tokyo, 135-8550 Japan; 3 Department of Pathology, Cancer Institute Hospital of Japanese Foundation for Cancer Research, 3-8-31 Ariake, Koto-Ku, Tokyo, 135-8550 Japan; 4 Department of Gastroenterology, Cancer Institute Hospital of Japanese Foundation for Cancer Research, 3-8-31 Ariake, Koto-Ku, Tokyo, 135-8550 Japan

**Keywords:** Rectal cancer, Deficient mismatch repair, Immune checkpoint inhibitor, Watch-and-wait approach, MSH6

## Abstract

**Background:**

Treating rectal cancer presents challenges due to postoperative complications and reduced quality of life (QOL). Recent evidence supports the watch-and-wait (WW) approach for patients with a clinical complete response (cCR) following preoperative treatment. In this report, we discuss a case of metastatic rectal cancer with deficient mismatch repair (dMMR) treated successfully with pembrolizumab.

**Case presentation:**

A 47-year-old male with dMMR rectal cancer and a single liver metastasis underwent treatment with pembrolizumab as neoadjuvant therapy. After 10 courses, the rectal lesion achieved cCR, prompting the selection of the WW approach. The liver metastasis showed significant shrinkage; however, the presence of a residual tumor was suspected, leading to a metastasectomy. A pathological complete response (pCR) was confirmed via histological examination. During a 24-month follow-up, there was no evidence of tumor regrowth, local recurrence, or distant metastasis.

**Conclusions:**

The WW strategy is increasingly accepted for patients achieving cCR after preoperative treatment. While pCR in dMMR rectal cancer patients treated with immune checkpoint inhibitors (ICIs) has been documented, accurately predicting pCR from imaging remains challenging. This case illustrates that integrating ICI therapy, surgical interventions, and the WW approach can effectively achieve both oncological safety and improved QOL in the treatment of dMMR metastatic rectal cancer.

## Background

Deficient mismatch repair (dMMR), a genetic anomaly, occurs in approximately 15% of colorectal cancer (CRC) cases in Western populations, compared to only 3.8–7% in Japanese individuals [1–4]. The prevalence of dMMR in rectal cancer is relatively low, estimated between 4% and 6.9% [5, 6]. Notably, the frequency of dMMR decreases with advancing stages of CRC, dropping to about 5% in metastatic cases [7]. CRC with dMMR has shown resistance to cytotoxic chemotherapeutic agents compared with proficient mismatch repair (pMMR) [8]. However, both locally advanced and metastatic rectal cancers with dMMR respond favorably to immune checkpoint inhibitors (ICIs) [9–11].

Persistent concerns about postoperative complications and a decline in quality of life (QOL) associated with surgery for lower rectal cancer have been documented [12]. The watch-and-wait (WW) strategy, applied to patients who achieve a clinical complete response (cCR) after preoperative treatment, has gained traction due to increasing evidence of its effectiveness [13].

Despite the presumed rarity of dMMR in metastatic rectal cancer, the favorable outcomes observed with ICIs suggest potential benefits such as avoiding surgery and preserving anal function. However, definitive treatment protocols have not been established due to insufficient evidence. Here, we present a case of metastatic rectal cancer treated with ICIs where primary rectal lesions that showed cCR were managed using the WW approach. Although multiple imaging modalities suggested a residual tumor, surgical resection of the metastatic liver lesion confirmed a pathologic complete response (pCR).

## Case presentation

**Fig. 1:**
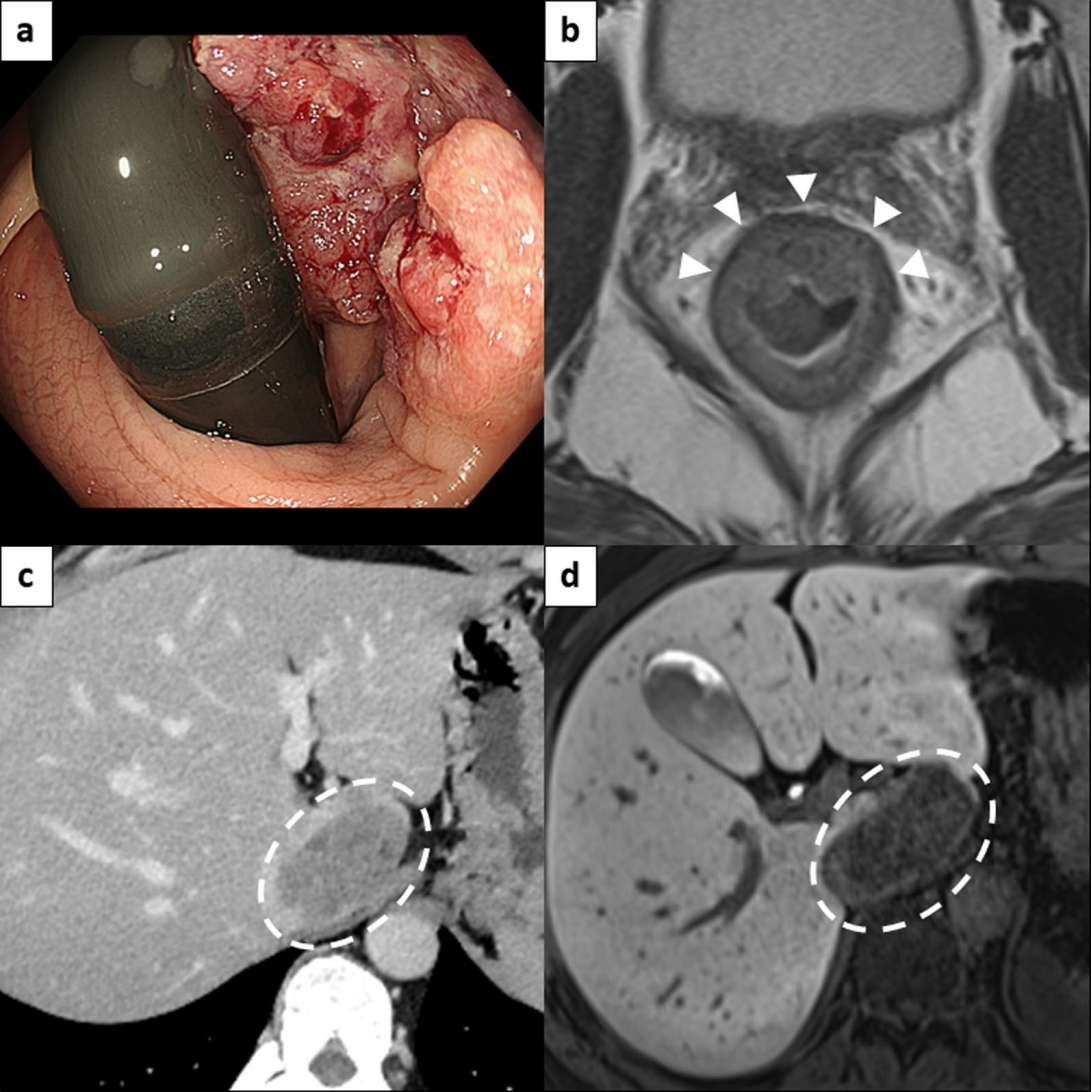
Imaging findings of primary and metastatic lesions before ICI therapy. **a** Inverted view of the endoscopic image at the rectum, showing a slightly elevated tumor located in the lower rectum. **b** Axial MRI sections of the rectal tumor, with arrowheads indicating a T2, EMVI-, CRM- rectal tumor. **c** CECT and **d** EOB-MRI of the liver, where the dashed line marks a 50 mm-sized solitary liver metastasis in the caudate

**Fig. 2:**
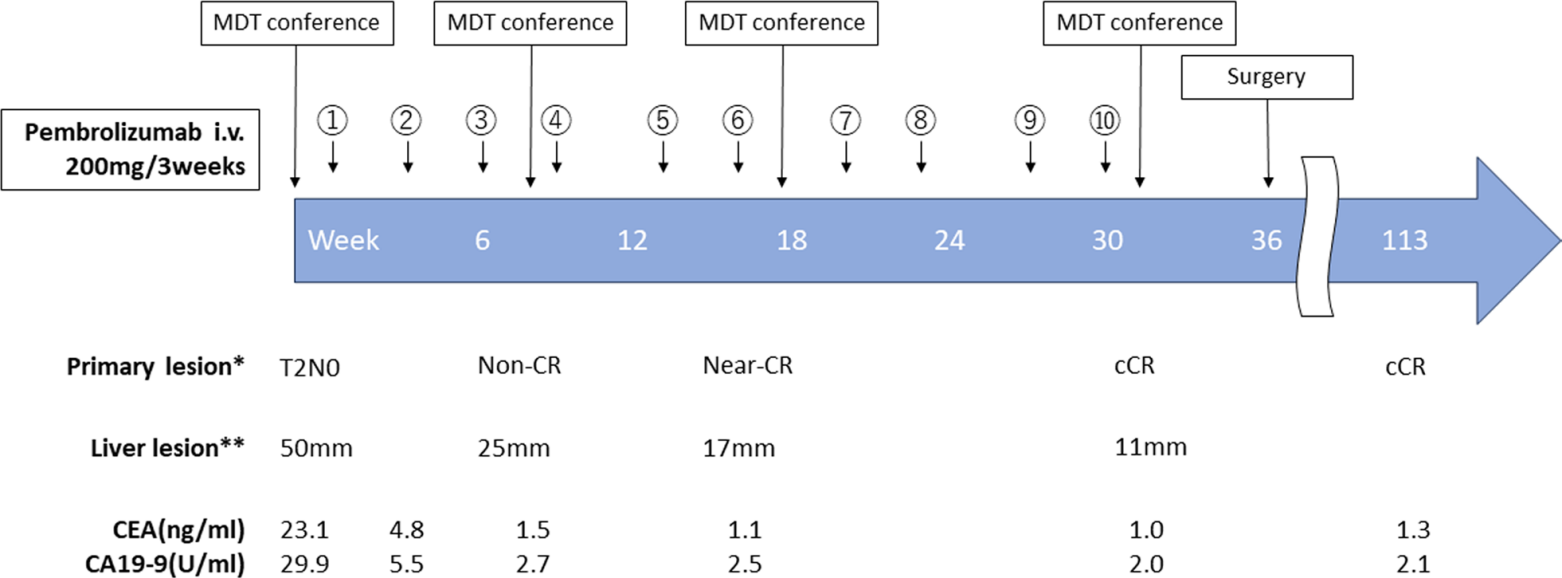
Timeline of treatment and evaluation. Day 0 marks the first course of pembrolizumab. i.v.: intravenous administration; MDT conference: multidisciplinary team conference; cCR: clinically complete response. *Assessment was conducted using multiple modalities, including endoscopy, CT, MRI, and digital rectal examination. **Tumor size was evaluated using gadoxetic acid (Gd-EOB-DTPA)-enhanced MRI

**Fig. 3:**
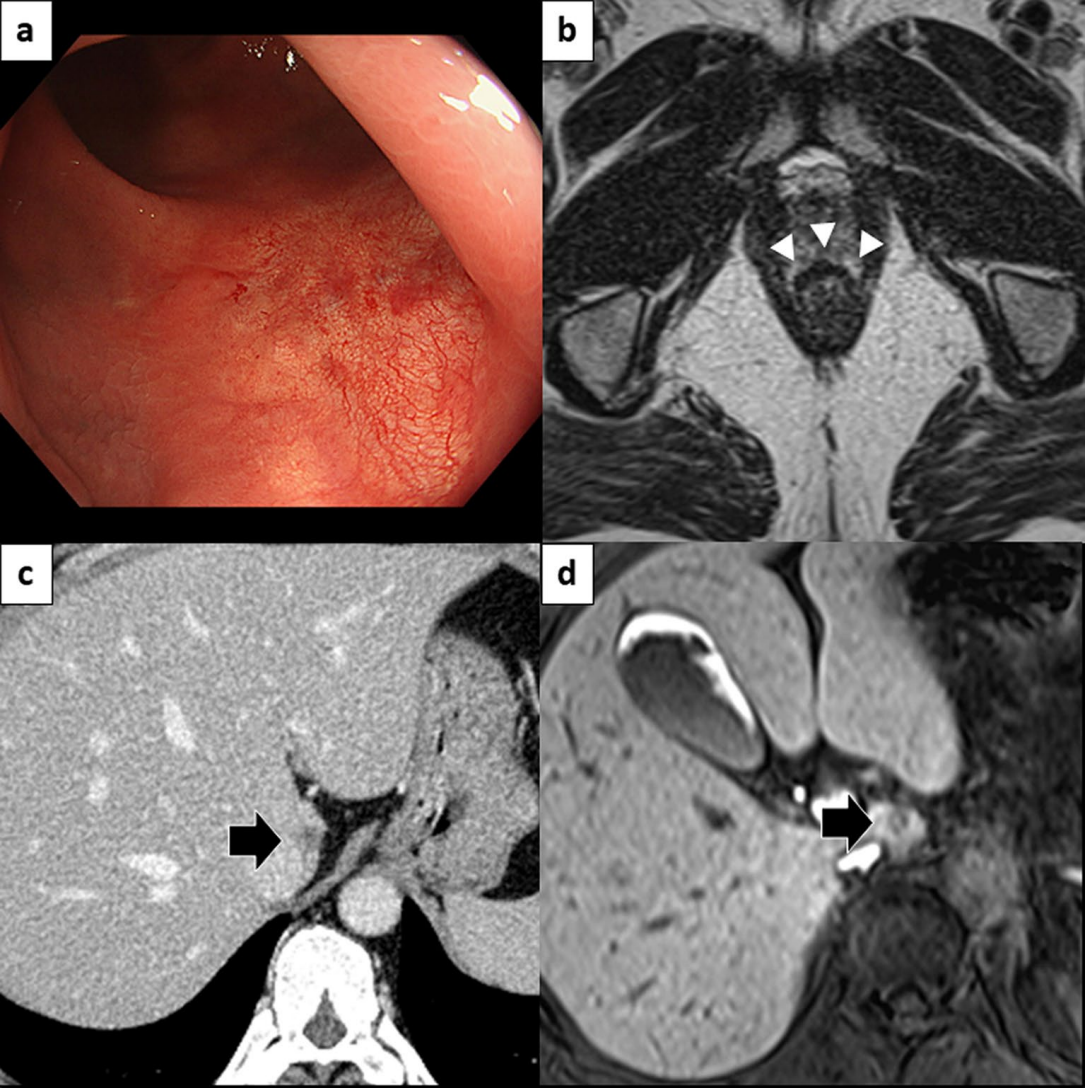
Imaging findings after ICI therapy. **a** The standard view of the endoscopic image at the lower rectum shows a flat scar without irregularities. **b** Axial MRI sections of the rectal area, with an arrowhead indicating the scar in the lower rectum with no evidence of residual tumor. **c** CECT and **d** EOB-MRI of the liver, with arrows pointing to a significantly shrunken liver metastasis

A 47-year-old male presented with bloody stools and was referred to our hospital. Colonoscopy revealed tumor in the lower rectum near the dentate line, which was pathologically diagnosed as moderately differentiated adenocarcinoma (Fig. 1a). Magnetic resonance imaging (MRI) staged the rectal tumor as T2, with no evidence of extramural vascular invasion (EMVI) or circumferential resection margin (CRM) involvement (Fig. 1b). Contrast-enhanced computed tomography (CECT) and gadoxetic acid (Gd-EOB-DTPA)-enhanced MRI identified a solitary 50 mm metastatic lesion in the caudate lobe of the liver (Fig. 1c, d). There was no obvious lymph node metastasis identified. Carcinoembryonic antigen (CEA) and carbohydrate antigen 19-9 (CA19-9) levels were 23.1 ng/mL and 29.6 U/mL, respectively. Immunohistochemical mismatch repair analysis showed loss of MSH6 with retained MLH1, MSH2, and PMS2 in the tumor. MSH6 sequencing identified c.3261del (p.Phe1088Serfs*2), leading to a diagnosis of Lynch syndrome, and genetic counseling was provided. Additionally, multiplex PCR confirmed MSI-high status. The KRAS gene had a G13D mutation, and BRAF was wild-type. The liver metastasis was classified as borderline resectable based on the institution’s criteria, which include having four or more liver metastases, any metastasis larger than 5 cm, or the presence of concomitant resectable extrahepatic metastases [14]. During the multidisciplinary team (MDT) conference, pembrolizumab was recommended as neoadjuvant therapy according to the MMR status. Figure 2 illustrates the timelines of treatments and assessments. After initiating treatment with pembrolizumab at a fixed dose of 200 mg administered intravenously every 3 weeks, an assessment after three courses showed shrinkage in both the liver and the primary lesion (non-CR), prompting consideration of surgical resection. However, considering the patient's preference for preserving anal function, the treatment continued. After a total of 10 courses of pembrolizumab, a cCR was achieved in the primary lesion (confirmed by digital rectal examination, MRI, and endoscopy), and the liver metastasis significantly reduced to 11 mm, without any immune-related adverse events (Fig. 3a–d). Tumor markers decreased to normal ranges, with CEA at 1.3 ng/mL and CA19-9 at 2.1 U/mL. The MDT recommended the WW approach for the rectal tumor and surgical intervention for the liver metastasis based on imaging that suggested the liver metastases were resectable. Additionally, their proximity to the inferior vena cava (IVC) was considered because of the risk that the tumors could become unresectable if they grew further. The rectal tumor was assessed as having achieved a cCR. The high likelihood of successful salvage surgery in the event of regrowth, coupled with the goal of preserving anal function, justified the decision for a WW approach. Subsequently, we performed a laparoscopic caudate resection with partial IVC resection for the liver metastasis, and the pathological examination confirmed a pCR. There was no evidence of rectal regrowth, recurrence, or metastasis of the primary tumor during the 24-month follow-up period without any treatment.

## Discussion

Surgical treatment remains a cornerstone of therapeutic strategies for lower rectal cancer; however, preserving anal or defecatory function poses challenges, potentially leading to a significant decrease in postoperative QOL. Balancing oncological thoroughness with quality of life remains a continual dilemma. Increasing evidence supports the utility of the WW approach for patients with advanced lower rectal cancer who have achieved a cCR after neoadjuvant chemoradiotherapy [15, 16]. Additionally, Garcia-Aguilar et al. reported that total neoadjuvant therapy (TNT) could potentially obviate the need for surgery in approximately half of the patients with locally advanced lower rectal cancer, specifically in those with pMMR [17]. Meanwhile, in patients with dMMR CRC, 60–79% who underwent primary tumor resection after preoperative ICI treatment achieved a pCR, and 50% of those who underwent metastasectomy achieved a pCR in metastatic lesions[18–20]. In this case, we opted for the WW approach for the primary rectal lesion after achieving a cCR. For the liver metastasis, where imaging indicated a residual tumor, we chose surgical intervention, which resulted in a pCR. While recent reports on the WW strategy for locally advanced rectal cancer after treatment with ICIs raise expectations of its effectiveness, there are no reported experiences of combining surgery and WW for metastatic rectal cancer [9, 21].

Evaluating the CR to ICIs using radiographic assessments is complex. Further, differentiating between tumor cells, infiltrating immune cells, mucin, and necrotic tissue complicates the evaluation [11]. Additionally, unique phenomena such as pseudo-progression have been reported, where the tumor appears to enlarge following treatment; however, this enlargement often indicates an effective response to therapy [22]. It should be noted that in this case, if the pCR of the liver lesion had been predicted before surgery, the WW approach might have been a viable option for liver metastasis as well. Compared to rectal lesions, which can be comprehensively assessed using methods such as endoscopy, CT, MRI, and digital rectal examination, the evaluation of metastatic lesions is limited to fewer modalities, such as CT, MRI, and ultrasound. This highlights the limitations in accurately determining CR for metastatic lesions. Developing methods that can accurately predict pCR is highly desirable. Several biomarkers, including circulating tumor DNA (ctDNA), have been suggested to better reflect the true response compared to radiographic evaluations [23]. However, their clinical application has not yet been widely adopted, and resection is inevitable when a residual tumor is clearly evident on imaging. Nonetheless, as demonstrated in this case, the impact on postoperative QOL after partial hepatectomy is often not as severe as that after lower rectal surgery.

The WW approach following ICI treatment involves significant uncertainties regarding oncological safety, the establishment of optimal follow-up protocols, and therapeutic choices for tumor regrowth. Therefore, caution is crucial in making these decisions, and it is essential to provide patients with a thorough explanation and obtain their informed consent. Nevertheless, given the characteristics of ICIs mentioned above, we believe that our chosen strategy of surgical intervention for liver metastases with residual tumors, coupled with a WW approach for primary sites where cCR is achieved, is a viable option in the treatment strategy for metastatic rectal cancer.

In this case, a loss of MSH6 with MSI-high status was observed in the tumor tissue, and germline mutation testing confirmed Lynch syndrome. *MSH6* accounts for approximately 10% of Lynch syndrome cases, which is less frequent than *MLH1* or *MSH2* [24]. CRC with loss of MSH6 has been reported to exhibit different characteristics compared to loss of MLH1 or MSH2. While dMMR CRCs are more common on the right side of the colon, loss of MSH6 is more frequent on the left side [25]. In particular, 25% of tumors associated with MSH6 develop in the rectum, compared to 5% for MLH1 or MSH2 tumors [26]. It has also been reported that loss of MSH6 is expected to display lower instability, and MSS status with loss of MSH6 is the most common discordance [27, 28]. In *MSH6* mutation carriers, the risk for CRC was lower than in those with *MLH1* or *MSH2* mutations [29]. There is limited data on the effectiveness of ICIs for the loss of MSH6, although this case showed a remarkable effect, the response to ICI treatment for MSH6 may be lower than for other mutations considering the lower instability. It has been reported the efficacy of ICIs varies depending on different MMR alterations, and there are cases where ICIs were ineffective in MSS CRC with MSH6 loss [30, 31]. Further accumulation of data is warranted to clarify these observations.

## Conclusions

This case demonstrates the potential for achieving both oncological safety and QOL by effectively integrating ICI therapy, surgical intervention, and the WW approach in the treatment of metastatic dMMR rectal cancer.

## Data Availability

Not applicable.

## Abbreviations

dMMR: Deficient mismatch repair
CRC: Colorectal cancer
pMMR: Proficient mismatch repair
ICIs: Immune checkpoint inhibitors
QOL: Quality of life
WW: Watch-and-wait
cCR: Clinical complete response
pCR: Pathological complete response
MRI: Magnetic resonance imaging
EMVI: Extramural vascular invasion
CRM: Circumferential resection margin
CECT: Contrast-enhanced computed tomography
CEA: Carcinoembryonic antigen
CA19-9: Carbohydrate antigen 19-9
MDT: Multidisciplinary team
IVC: Inferior vena cava
